# Metamaterial-enabled arbitrary on-chip spatial mode manipulation

**DOI:** 10.1038/s41377-022-00859-9

**Published:** 2022-06-01

**Authors:** Jinlong Xiang, Zhiyuan Tao, Xingfeng Li, Yaotian Zhao, Yu He, Xuhan Guo, Yikai Su

**Affiliations:** State Key Laboratory of Advanced Optical Communication Systems and Networks, Department of Electronic Engineering, Shanghai Jiao Tong University, Shanghai, 200240 China

**Keywords:** Metamaterials, Optical manipulation and tweezers, Integrated optics

## Abstract

On-chip spatial mode operation, represented as mode-division multiplexing (MDM), can support high-capacity data communications and promise superior performance in various systems and numerous applications from optical sensing to nonlinear and quantum optics. However, the scalability of state-of-the-art mode manipulation techniques is significantly hindered not only by the particular mode-order-oriented design strategy but also by the inherent limitations of possibly achievable mode orders. Recently, metamaterials capable of providing subwavelength-scale control of optical wavefronts have emerged as an attractive alternative to manipulate guided modes with compact footprints and broadband functionalities. Herein, we propose a universal yet efficient design framework based on the topological metamaterial building block (BB), enabling the excitation of arbitrary high-order spatial modes in silicon waveguides. By simply programming the layout of multiple fully etched dielectric metamaterial perturbations with predefined mathematical formulas, arbitrary high-order mode conversion and mode exchange can be simultaneously realized with uniform and competitive performance. The extraordinary scalability of the metamaterial BB frame is experimentally benchmarked by a record high-order mode operator up to the twentieth. As a proof of conceptual application, an 8-mode MDM data transmission of 28-GBaud 16-QAM optical signals is also verified with an aggregate data rate of 813 Gb/s (7% FEC). This user-friendly metamaterial BB concept marks a quintessential breakthrough for comprehensive manipulation of spatial light on-chip by breaking the long-standing shackles on the scalability, which may open up fascinating opportunities for complex photonic functionalities previously inaccessible.

## Introduction

**Fig. 1:**
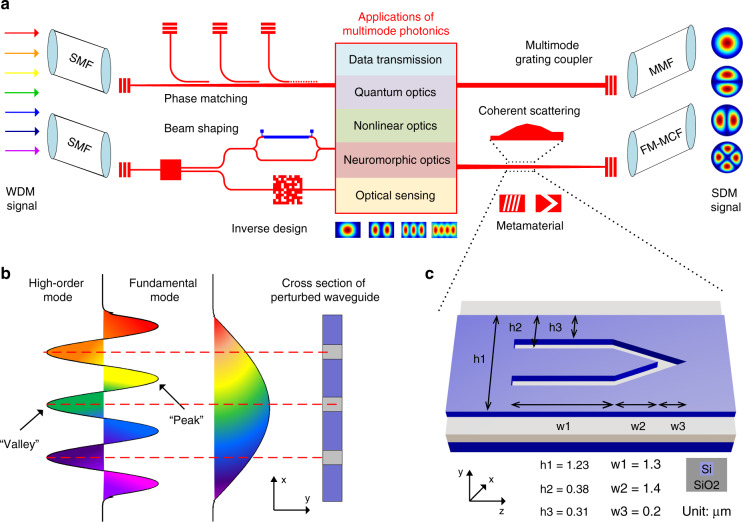
Integrated multimode photonics and metamaterial building block. **a** On-chip mode manipulation techniques and multimode photonic application scenarios. WDM wavelength-division multiplexing, SMF single-mode fiber, MMF multimode fiber, FM-MCF few-mode multicore fiber, SDM space-division multiplexing. **b** Dielectric perturbations are located at the “peaks” or “valleys” in the transverse mode profile of high-order mode to maximize the mode overlap integral. **c** Schematic view of the metamaterial TE_0_-TE_2_ building block (BB), exploiting fully etched dielectric slots on a silicon waveguide

The internet data traffic has increased by more than a thousandfold in the last 20 years. Spatial-division multiplexing (SDM), employing different spatial orthogonal modes, has been widely explored to tackle the upcoming forecasted “capacity crunch” in optical fiber communications^1,2^. Ultra-high-capacity data transmission has been demonstrated using single-mode multicore fibers (MCFs)^3^, multimode fibers (MMFs)^4^ as well as their hybrid combination of few-mode multicore fibers (FM-MCFs)^5^. Besides, efficient mode multiplexing in free space has been achieved with phase plates and multi-plane light conversion^4^. As illustrated in Fig. 1a, through introducing the spatial-mode-parallelism dimension to silicon photonic integrated circuits (PICs), mode-division multiplexing (MDM) can significantly scale the bandwidth density of on-chip interconnects^6,7^, and also has great potential in MMF communications where the MMF is excited directly by the multimode waveguide via a multimode grating coupler^8^. Moreover, mode-selective manipulation has greatly promoted the development of diverse information processing fields ranging from performance-enhanced optical sensing^9,10^, neuro-inspired photonic computing^11^, to novel nonlinear^12^, and quantum optics devices^13,14^. For instance, the coupling of spatial modes with other degrees of freedom (polarization, time, frequency, etc.) allows one to encode and process quantum information in higher dimensions, thus giving rise to more efficient logic gates and noise resilient communications^13,14^. Besides, a neuro-inspired photonic reservoir for high-speed chaotic time series prediction has been realized by utilizing the complex interference between multiple guided modes^11^. Moreover, the phase-matching condition for four-wave mixing can be satisfied in the visible regime through the dispersion engineering of high-order waveguide modes, which opens an interesting wavelength window for nonlinear applications^15^.

The essential foundation of multimode silicon photonics is on-chip spatial mode manipulation, which has attracted tremendous research efforts over the past decade. Traditional techniques schematically shown in Fig. 1a, i.e., phase matching^16,17^, beam shaping^18,19^, and coherent scattering^20,21^, all exhibit compromised performance in terms of excess losses (ELs), modal crosstalk (CT), fabrication tolerance, and device footprints. More specifically, although extensive mode (de)multiplexers have been implemented with cascaded asymmetric directional couplers (ADCs)^16^ as well as subwavelength grating couplers (SWGs)^17^, the phase-matching condition can hardly be satisfied and becomes extremely sensitive to fabrication imperfections for high-order modes, due to the large contrast in dispersion slopes between the bus waveguide and the access waveguide. While the beam shaping method generally needs complicated designs of mode splitting, phase shifting, and mode combining^18,19^. Moreover, long optical paths are required to introduce a phase difference of π between adjacent branches. As for the coherent scattering approach, the widely used inverse design method has enabled efficient mode conversion within ultra-compact footprints^22,23^ by exploring the full design parameter space of photonic devices with arbitrary topologies^24^. However, the performance is heavily dependent on the numerical optimization algorithms. Besides, the generated irregular nanostructures inevitably demand high fabrication accuracy owing to the tiny feature size.

Metamaterials, consisting of optical antenna arrays on the subwavelength scale, can provide advanced control of the optical wavefronts in both free space and integrated waveguides, leading to numerous applications from high-efficiency holograms, ultrathin cloaks, to analog mathematical computing, nonlinear and topological photonics^25–27^. More recently, metamaterials have been introduced to silicon PICs as a competitive alternative to control the light propagation on chip, and are particularly attractive in two aspects. On the one hand, metamaterials are capable of scattering guided modes to new wavevectors within a propagation distance of only several times the wavelength, thus significantly reducing the device footprints^28^. Meanwhile, broadband mode manipulation can be guaranteed due to an inverse relation between the device dimension and working bandwidth^29^. Previously, a diversity of mode converters with impressive performance have been reported by imposing partially or fully etched nanostructures on metamaterial waveguides^30–33^. However, mode manipulation up to the fifth order still remains unachievable.

It should be noted that the abovementioned techniques all face fundamental limitations on scalability. First, these approaches are typically specific mode-order-oriented, which means each single-mode operator requires a pre-determined structure selection based on analytic theory and intuition, followed by considerable iterations to optimize geometrical parameters, thus inevitably leading to a long development time and huge trial-and-error costs. Second, arbitrary high-order mode manipulation is inherently not supported, restricted by either the working principle itself, e.g., the coherent scatting approach, or the available fabrication technologies, e.g., the phase-matching method. Consequently, mode converters reported so far have mainly been constrained to low-order mode cases.

To this end, we develop a universal, simple yet efficient design framework to implement arbitrary on-chip mode conversion and mode exchange simultaneously, based on the novel topological metamaterial building block (BB) concept. Dielectric perturbations are rationally engineered on metamaterial waveguides to realize the straightforward beam shaping principle, which induce strong energy coupling between guided modes of interest within an ultra-compact conversion region. Our general strategy features a user-friendly specification-oriented design, whereby the user simply defines the desired order of mode manipulation, and arbitrary even-order and odd-order mode operators can be directly determined by programming the topological arrangement of multiple primitive TE_0_-TE_2_ BBs with predefined mathematical formulas. As such, uniform good performance with the ELs below 1.5 dB and the CT lower than −8 dB (−12.5 dB assisted with a taper) from 1500 to 1600 nm has been achieved for arbitrary high-order mode operators in numerical simulations, which is further validated by sufficient experimental results. To benchmark the extraordinary scalability of the metamaterial BB frame, we have experimentally implemented record high-order mode manipulation up to the twentieth in silicon nanophotonics, to the best of our knowledge. As a proof-of-concept demonstration of the possible multimode applications for the metamaterial BB framework, high-speed data transmission of 8-channel 16-quadrature amplitude modulation (16-QAM) signals is successfully verified at a symbol rate of 28 gigabauds (GBaud) and an aggregate data rate of 813 Gb/s with bit error rates (BERs) under the 7% forward error correction (FEC) threshold of 3.8 × 10^−3^.

## Results

### Working principle and metamaterial building block

The propagation of electromagnetic waves in a perturbed metamaterial waveguide can be approximately described by the classical coupled-mode theory (CMT)^34,35^. Assuming that guided modes propagate along the z direction, the electric field distribution can be represented by a superposition of all the supported eigenmodes:

1 $$E\left( {x,y,z} \right) = \mathop {\sum}\nolimits_m {A_m(z)\psi _m(x,y)e^{ - j\beta _mz}}$$

where *m* is the mode subscript, *A*_*m*_(*z*) is the amplitude, *β*_*m*_ is the propagation constant, and *ψ*_*m*_(*x,y*) is the electric mode profile of the *m*th-order eigenmode, respectively. Due to the mode coupling, *A*_*m*_(*z*) is dependent on the propagation distance and can be derived from the CMT equations:

2 $$\frac{d}{{dz}}A_m(z) = - j\mathop {\sum}\nolimits_n {\kappa _{mn}(z)A_n(z)e^{ - j(\beta _n - \beta _m)z}}$$

where *κ*_*mn*_ is the coupling coefficient between the *m*th-order mode and the *n*th-order mode, and defined as:

3 $$\kappa _{mn}\left( z \right) = \frac{\omega }{4}{\int\!\!\!\!\!\int} {\psi _m^ \ast (x,y){{\Delta }}\varepsilon (x,y,z)\psi _n(x,y)dxdy}$$

where * denotes the complex conjugate, Δ*ε*(*x,y,z*) is the refractive index perturbation. To realize efficient on-chip mode conversion, metamaterial waveguides should provide the necessary momentum compensation for the wavevector matching between guided modes of interest. Besides, the spatial distribution of Δ*ε*(*x,y,z*) needs to be carefully engineered to maximize the “field overlap” integral ${\int\!\!\!\!\!\int}{\int\!\!\!\!\!\int} {\psi _m^ \ast (x,y){{\Delta }}\varepsilon (x,y,z)\psi _n(x,y)dxdy}$ in Eq. (3) coupling coefficient. Therefore, dielectric perturbations are commonly introduced to the regions where “peaks” or “valleys” are located in the transverse field profile of the high-order mode, as shown in Fig. 1b. In former studies, metamaterial mode converters have been obtained by periodically varying the refractive index distribution along the propagation direction^31,35,36^; however, there exists a huge gap for the effective medium theory mapping^37^ between the ideal index profile and the physical dielectric structure, constrained especially by the fabrication requirements. Besides, mode manipulation has also been realized by modifying the supermode field profiles of SWG metamaterial waveguides^30^, which suffers the time-consuming mode-order-oriented optimization process.

Previously, we have demonstrated that a single dielectric slot can function as a power splitter and a phase shifter simultaneously^33^, which inspires the implementation of beam shaping principle completely with metamaterial BBs consisting of fully etched dielectric slots (more information about the origin of metamaterial BBs is provided in Supplementary Note 1). Considering the symmetry property of electric field profiles of eigenmodes, the TE_0_-TE_2_ metamaterial BB^38^ is designed with a symmetric arrow-like shape, as depicted in Fig. 1c. The two straight arms help to separate the multimode waveguide into three single-mode channels and well confine the electric field to each field “peak”, while the followed V-shaped groove induces proper phase differences between the three quasi-TE_0_ beams and combines them into the desired output mode. In this way, strong energy coupling between involved modes is obtained within an ultra-compact footprint of 1.23 × 2.7 μm^2^. Moreover, the processes of mode splitting, phase shifting, and mode combining are independent and reciprocal on the input mode. As a result, the metamaterial BB inherently supports the functionalities of both mode conversion and mode exchange, which has not been reported in most existent literature (a performance comparison of on-chip mode converters is provided in Supplementary Note 6).

**Fig. 2:**
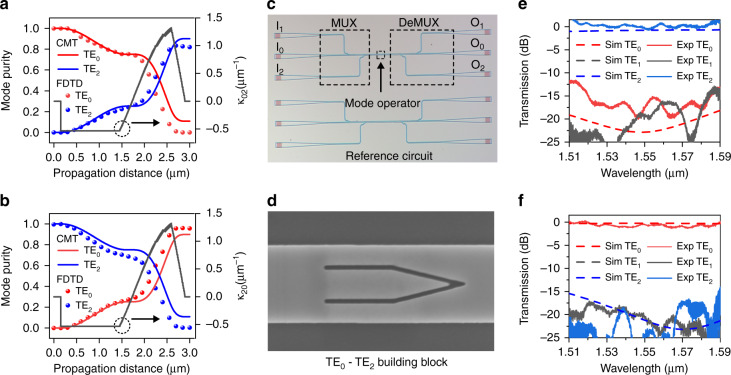
Simulated and experimental results of the metamaterial BB. **a**, **b** Calculated mode purity and coupling coefficients as a function of the propagation distance for the TE_0_ and TE_2_ input mode, respectively. **c** Microscope picture of the fabricated photonic integrated circuit (PIC) consisting a 3-channel mode multiplexer (MUX), a TE_0_-TE_2_ mode operator, and a 3-channel mode demultiplexer (DeMUX). **d** Scanning electron microscope (SEM) image of a fabricated metamaterial TE_0_-TE_2_ BB. **e**, **f** Simulated and measured mode conversion efficiency for the TE_0_ and TE_2_ input mode, respectively. The crosstalk from the TE_1_ mode is below −25 dB in simulation, thus not shown in both figures

Figure 2a, b presents the simulated mode evolution processes and coupling coefficients for the TE_0_-to-TE_2_ and TE_2_-to-TE_0_ mode conversion, respectively. It can be seen that the input mode is gradually converted to the target output mode within a short propagation distance of only 2.7 μm (from 0.15 to 2.85 μm), and the calculated mode purity with the CMT model matches well with the 3D ﻿finite-difference time-domain (3D-FDTD) simulation results. Besides, the coupling coefficient, which is roughly analog to a sinusoidal function, experiences a transition from negative to positive values, thus ensuring the entire constructive contribution to the desired conversion^35^. To experimentally verify the TE_0_-TE_2_ metamaterial BB, we have fabricated a silicon PIC consisting of a mode multiplexer with three input ports I_0_–I_2_, a TE_0_-TE_2_ mode operator, and a mode demultiplexer with three output ports O_0_–O_2_, as shown in Fig. 2c. An extra PIC composed of two back-to-back (B2B) mode (de)multiplexers is also fabricated on the same chip to normalize the transmission spectra of the device. The designs of mode (de)multiplexers are based on traditional ADCs structures. Figure 2d shows the scanning electron microscope (SEM) image of a TE_0_-TE_2_ metamaterial BB. Both the simulated and measured mode manipulation efficiency are shown in Fig. 2e, f for comparison. In simulations, the ELs are below 1.3 dB and the CT is lower than −15 dB from 1510 to 1590 nm in both scenarios. It should be noted that the crosstalk from the TE_1_ mode is extremely low (<−25 dB) and not shown in both plots. The measured ELs for the TE_0_-to-TE_2_ mode conversion are below 0.74 dB with the CT lower than −12.75 dB in the wavelength range of 1510–1590 nm. For the TE_2_-to-TE_0_ mode conversion, the measured ELs are below 1.4 dB with the CT lower than −13.98 dB within the same wavelength band. Especially, in the wavelength range of 1520–1580 nm, the measured ELs are lower than 1.4 dB and the CT is below −15 dB for both input mode cases. Overall, the experimental results agree well with the simulation results.

### Arbitrary high-order mode operator

The beam shaping principle provides a straightforward route to control arbitrary high-order modes; however, its great potential has been significantly constrained by the complicated designs based on the traditional Mach–Zehnder interferometer architecture. Here we propose, for the first time, a practicable universal implementation scheme by programming multiple metamaterial BBs in a simple parallel layout. Taking into consideration the symmetry property of electric field distributions of eigenmodes, the even-order and odd-order mode operators are addressed separately.

**Fig. 3:**
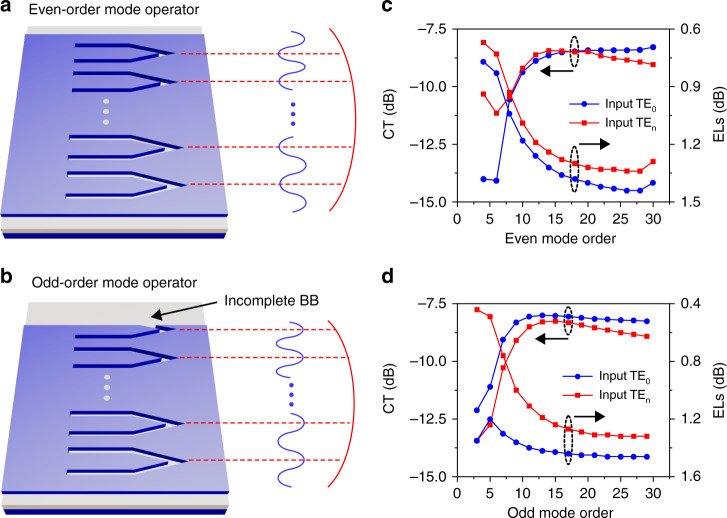
Design and simulated results for arbitrary high-order mode operator. **a**, **b** The 3D view for the even-order and odd-order mode operator, respectively. **c**, **d** The corresponding calculated excess losses (ELs) and crosstalk (CT) in the wavelength range from 1500 to 1600 nm

We first detail the metamaterial implementations of even-order mode operators. As shown in Fig. 3a, there exists *N*/2+1 in-phase “peaks” and *N*/2 anti-phase “valleys” in the transverse electric field profile of the TE_*N*_ mode (*N* is an even number). To realize the TE_0_-TE_*N*_ mode manipulation with the beam shaping technique, *N*/2 metamaterial BBs, containing *N* dielectric slots in total, are needed to divide the multimode waveguide into *N*+1 single-mode channels. Besides, in order to maximize the coupling coefficient between the two involved modes, the metamaterial BBs are engineered to exactly point at the positions of field “valleys” (the implementation scheme of placing the metamaterial BBs at the field “peaks” is discussed in Supplementary Note 3). The symmetric geometry of the even-order mode operator is now determined by only two parameters, i.e., the central distance between adjacent metamaterial BBs *w*_*d*_ and the waveguide width *w*_even_. For simplicity, *w*_*d*_ is assumed to be constant and *w*_even_ is expressed with a uniform formula:

4 $$w_{{\rm{even}}} = w_d\frac{N}{2} + 2w_{{\rm{extra}}}$$

where *w*_extra_ is an extra waveguide width applied on both sides to better confine the mode field. The optimized results from 3D-FDTD simulations are *w*_*d*_ = 0.92 μm and *w*_extra_ = 0.19 μm, respectively.

For the implementation of the TE_0_-TE_*N*_ odd-order mode operator, there is only one possible layout arrangement of metamaterial BBs, given the fact that the electric mode profile of the TE_*N*_ mode possesses central symmetry with the same number of field “peaks” and “valleys”. As shown in Fig. 3b, (*N* + 1)/2 metamaterial BBs are engineered to point at the (*N* + 1)/2 field “peaks” of the TE_*N*_ mode, and the metamaterial BB closest to the waveguide edge partially extends beyond the original waveguide region, as indicated by the “incomplete BB”. From the perspective of geometry structure, it is natural and reasonable to consider that the TE_0_-TE_*N*_ odd-order mode operator can be obtained by directly truncating the TE_0_-TE_*N*+1_ even-odd mode operator with a fixed width *w*_offset_. As a result, the waveguide width *w*_odd_ can be determined by:

5 $$w_{{\rm{odd}}} = {{d}}\frac{{N + 1}}{2} + 2w_{{\rm{extra}}} - w_{{\rm{offset}}}$$

where *w*_offset_ is the width of the truncated part, and the optimized value is 0.46 μm.

We quantitatively evaluate the mode manipulation efficiency of the universal metamaterial mode operators, and the simulated ELs and CT across a broad wavelength range from 1500 to 1600 nm are summarized in Fig. 3c, d, respectively. As the mode order increases, both the ELs and CT first increase and eventually converge to a small oscillation range. Explicitly, the metamaterial mode operator shares uniform performance with the ELs lower than 1.5 dB and CT below −8 dB over a 100 nm wavelength span. Besides, the major contribution of CT comes from the related TE_0_, TE_2_, TE_*N*–2_, and TE_*N*_ mode, which can be largely attributed to their similar electric mode profiles of the same symmetry. It should be noted that the simple mathematically defined layout arrangement of metamaterial BBs is intended to offer an initial design prototype for arbitrary high-order mode manipulation, and the conversion efficiency can be remarkably enhanced by further optimizing the geometrical parameters for a specific mode. For example, the CT value of the TE_0_-to-TE_*N*_ mode conversion can be improved from −8 to −12.5 dB by adding a properly designed taper (see Supplementary Note 8). Moreover, the device footprint of the metamaterial mode operator is only linearly dependent on the mode order with a constant length of 2.7 μm, hence making it possible for high integration density. Furthermore, it also features excellent thermal stability and good fabrication tolerance to the perturbation width variation of ±15 nm (see Supplementary Note 4).

**Fig. 4:**
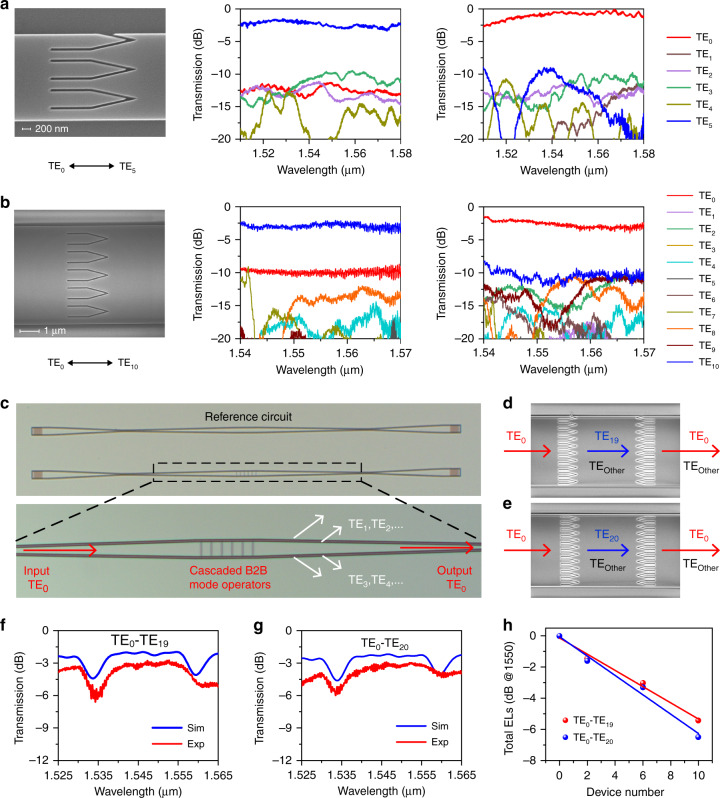
Experimental results of high-order mode operators. **a** Displayed from left to right are the SEM image of a fabricated TE_0_-TE_5_ mode operator (left), the measured mode conversion efficiency when the input is the TE_0_ mode (middle), and the TE_5_ mode (right). **b** Similar results corresponding to the TE_0_-TE_10_ mode operator. **c** Microscope pictures of the fabricated PIC consisting of a mode spot size converter, multiple cascaded B2B mode operators, and a high-order mode filter. **d**, **e** SEM images for the zoom-in view of two B2B high-order mode operators. **f**, **g** Measured transmission spectra for six cascaded B2B TE_0_-TE_19_ and TE_0_-TE_20_ mode operators, respectively. **h** The total ELs are linearly dependent on the device number

We have fabricated a series of high-order mode operators to experimentally verify the metamaterial BB concept. Mode (de)multiplexers consisting of cascaded ADCs and SWGs are utilized to characterize the performance of devices. Figure 4a, b presents the SEM images of fabricated TE_0_-TE_5_ and TE_0_-TE_10_ mode operators and their measured mode manipulation efficiency, respectively (more experimental results are provided in Supplementary Note 5). In the wavelength range from 1520 to 1580 nm, the TE_0_-TE_5_ mode operator can convert the TE_0_ input mode into the TE_5_ output mode with the ELs lower than 3 dB and CT below −7.2 dB, and the major crosstalk is original from the TE_3_ mode. Meanwhile, it can also convert the TE_5_ input mode into the TE_0_ output mode with the ELs lower than 1.7 dB and CT below −8.3 dB. Moreover, the TE_0_-TE_10_ mode operator exhibits similar performance with the ELs lower than 3.8 dB and CT below −7 dB across the wavelength band from 1540 to 1570 nm in both input cases. In general, the measured results are comparable to the numerical simulations.

The mode-by-mode characterization successfully validates the high-order mode manipulation capacity of the proposed metamaterial BBs. It should be noted that the performance degradation suffered by high-order mode (de)multiplexers actually poses the greatest challenge during the whole performance assessment process. Although record high-order mode (de)multiplexer has been experimentally demonstrated up to the 15th^39^, the critical phase-matching condition can hardly be well satisfied for all mode channels simultaneously owing to fabrication imperfections. In order to validate the extraordinary scalability of the BBs-based design framework, we characterize the performance of high-order mode manipulation (>15th) by directly measuring the total ELs of 2, 6, and 10 cascaded B2B mode operators, which has already been verified to be a useful and effective method^30,40^. As shown in Fig. 4c, the designed PIC consists of a taper-based mode spot size converter, multiple cascaded B2B mode operators, and a taper-based high-order mode filter. The TE_0_ input mode from the single-mode waveguide first gradually evolves into the fundamental mode of the multimode waveguide, then repeatedly experiences the mode conversion process of TE_0_-to-TE_*n*_ and TE_*n*_-to-TE_0_ several times, as explained in Fig. 4d, e, and finally transmits through the mode filter with almost no loss. Since the high-order modes generated in the conversion region are all filtered out, the total ELs measured at the output port directly represent the mode manipulation efficiency. The measured transmission spectra for 6 cascaded B2B TE_0_-TE_19_ and TE_0_-TE_20_ mode operators are presented in Fig. 4f, g, respectively (More information is provided in Supplementary Note 5). It should be noted that the cascaded configuration of mode operators forms a Fabry–Perot-like cavity, resulting in periodic resonances in the transmission spectra. It is clear that the general trends of experimental results agree with the simulation results, particularly for the positions and shapes of resonance dips. Due to the accumulation of fabrication errors, the measured total ELs increase almost linearly with the device number, as shown in Fig. 4h. The average ELs for the TE_0_-TE_19_ and TE_0_-TE_20_ mode conversion are estimated to be ~0.53 and ~0.63 dB, respectively.

### High-speed data communication

**Fig. 5:**
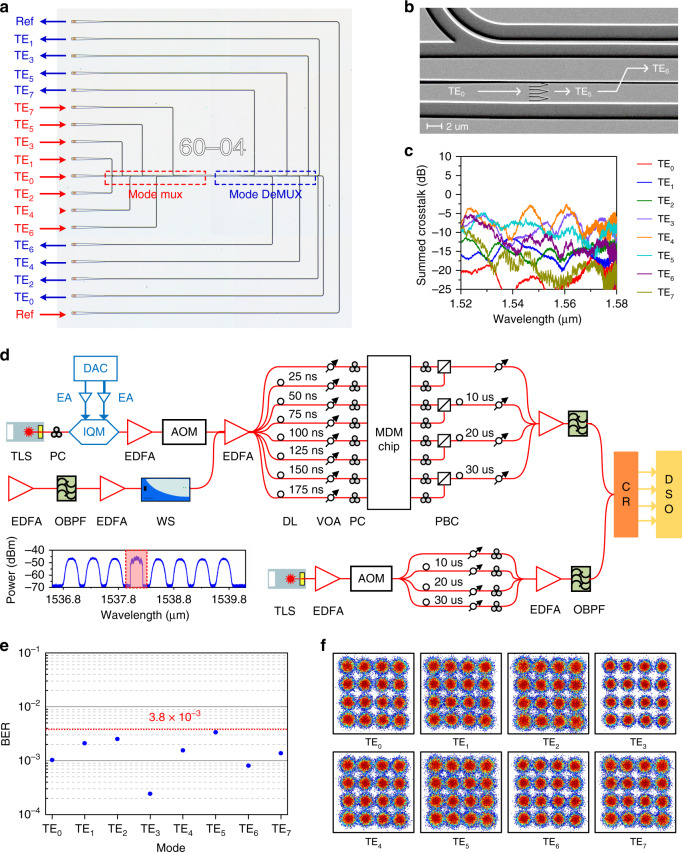
Eight-mode data transmission of 28-GBaud 16-QAM signals. **a** Microscope image of the fabricated 8-channel MDM PIC. **b** SEM image of the two-stage multiplexer for the TE_6_ mode, consisting of a metamaterial TE_0_-TE_5_ mode operator and a TE_5_-TE_6_ ADC. **c** Measured summed modal crosstalk from the other seven channels for each output port. **d** Experimental setup for high-speed transmission of 28-GBaud 16-QAM signals. TLS tunable laser source, PC polarization controller, DAC digital-analog converter, EA electronic amplifier, IQM in-phase/quadrature modulator, EDFA erbium-doped fiber amplifier, OBPF optical band pass filter, WS wave shaper, AOM acousto-optical modulator, DL delay line, VOA variable optical attenuator, PBC polarization beam combiner, CR coherent receiver, DSO digital storage oscilloscope. **e** Measured bit error rates (BERs) for eight channels. **f** Corresponding recovered constellation diagrams for eight modes

We take the high-speed data transmission scenario as a proof-of-principle application of the metamaterial-assisted universal multimode manipulation. An 8-channel BBs-based MDM circuit is fabricated on a silicon-on-insulator (SOI) wafer, as shown in Fig. 5a. The device consists of a mode multiplexer with eight input ports (marked in red), a multimode waveguide of 60 μm, and a mode demultiplexer with eight output ports (marked in blue). Except for the TE_1_ mode directly multiplexed with a TE_0_-TE_1_ ADC, the other six high-order modes are obtained with two-stage mode conversion. As illustrated in Fig. 5b, the input TE_0_ mode will first be converted to a middle high-order mode through a metamaterial mode operator, which is then coupled into the final desired mode of the bus waveguide with a carefully designed TE_*N*_-to-TE_*N*+1_ ADC. To characterize the MDM chip, the summed crosstalk from the other seven channels is measured for each output port. As shown in Fig. 5c, the CT ranges from −7.6 to −26.2 dB at 1540 nm for all mode channels. Then, we successfully demonstrate the high-speed data transmission of 28-GBaud 16-QAM optical signals via the 8-channel MDM circuit. The experimental setup is shown in Fig. 5d and described in detail in the method part. The calculated BERs for eight modes are shown in Fig. 5e, with all below the 7% FEC limit of 3.8 × 10^−3^. Besides, the corresponding recovered constellation diagrams are also presented in Fig. 5f, which indicates a good signal quality for all eight channels, and the on-chip data transmission aggregate data rate is measured to be 813 Gb/s.

Following the same development path of SDM fiber communications, the key technology to enhance the channel capacity of on-chip MDM optical interconnects is to manipulate as many waveguide modes as possible. For example, experimental results have shown an increasing single wavelength net capacity of 192 Gb/s, 1.23 Tb/s, and 1.51 Tb/s in MDM transmission of 3-mode PAM-4 signals^41^, 11-mode 16-QAM signals^42^, and 16-mode 16-QAM signals^39^, respectively. The metamaterial BBs make it possible to efficiently manipulate record high-order modes in practice, thus significantly boosting the transmission capacity and bandwidth efficiency of on-chip optical links. Moreover, once the multimode fiber-to-chip coupling techniques are improved in the future, the metamaterial-enabled arbitrary mode manipulation can play an important role in further combining the application scenarios of long-distance information transmission and on-chip data transmission together. It is worth mentioning that all necessary components for coherent communications have been demonstrated on integrated platforms, including narrow-linewidth laser sources^43^, high-speed in-phase/quadrature modulators (IQM)^44^, and high-speed photodetectors^45^. Besides, integrated coherent receiver^46^ and transmitters^47^ have also been experimentally reported. Therefore, it is promising to obtain fully integrated MDM communication systems of ultra-high spectral efficiency.

## Discussion

In conclusion, we have proposed a universal metamaterial-assisted BB framework to manipulate arbitrary on-chip spatial modes. The mathematically predefined topological arrangement of high-order mode operators allows user-friendly specification-oriented design process. As such, uniform good performance of low ELs, low CT, and broad bandwidth has been achieved in both simulations and experiments. Besides, record high-order mode manipulation up to the twentieth has been experimentally demonstrated to benchmark the excellent scalability of the metamaterial BBs. Furthermore, high-speed on-chip 8-channel MDM data transmission has been successfully verified with an aggregate data rate of 813 Gb/s (7% FEC). It is worth mentioning that the metamaterial BBs-based designs also feature compact footprints, eased fabrication process, and good fabrication tolerance. The presented generic mode manipulation approach represents critical progress towards advanced control of more physical dimensions of optic carriers, and the concept itself can be flexibly transferred to other waveguide platforms (InP, Si_3_N_4_, etc.) as well as other wavelength bands (O band, mid-infrared band, etc.). The fundamentals gained from our on-chip arbitrary spatial mode manipulation may provide inspiration for more versatile metamaterial-assisted BB designs and could promise a great breakthrough to boost the development of integrated quantum photonics, nonlinear photonics, and optical sensing.

## Materials and methods

### Device simulation

In the numerical simulations of CMT model, the transverse electric mode profiles of eigenmodes are obtained with the effective index method^48^, and the refractive index distribution of metamaterial structures is generated manually with *n*_*Si*_ = 3.476 and $n_{{\rm{SiO}}_2} = 1.444$. For the 3D-FDTD simulations (FDTD solutions, Lumerical), we define the metamaterial structures directly with the default material database, which has considered the material dispersion. And the simulation time is set to be 3000 fs, within which the auto-shutoff criteria can be satisfied. Besides, the discretization grid is automatically generated with a mesh accuracy index of 4, which provides a good tradeoff between accuracy, memory requirements and simulation time. Other settings remain the default values. Besides, the ELs and CT are defined as ${\rm{ELs}} = - 10{\rm{log}}_{10}\frac{{P_{{\rm{desired}}}}}{{P_{{\rm{input}}}}}$ and ${{{\mathrm{CT}}}} = {{{\mathrm{max}}}}\{ 10{\rm{log}}_{10}\frac{{P_{{\rm{other}}}}}{{P_{{\rm{desired}}}}}\}$, where *P*_input_, *P*_desired_, and *P*_other_ are the power of the input mode, the desired output mode, and the other interfering mode, respectively.

### Device fabrication

The devices are fabricated on an SOI wafer with a 220 nm top silicon layer on a 3 μm silicon dioxide layer. The designed patterns are first defined by the electron beam lithography system and then fully etched by using a single-step inductively coupled plasma dry etching. A 1-μm-thick silicon dioxide protection layer is deposited on top of the devices by plasma-enhanced chemical vapor deposition.

### Measurement setup

Grating couplers are used to interface the silicon waveguides and the single-mode fibers, with the coupling loss optimized to be 6.5 dB per facet. The input light from a continuous-wave tunable laser source (Keysight 81960A) is directly launched onto the chip after being polarized by a polarization controller, and the output light is monitored by an optical power meter (Keysight N7744A). For the mode-by-mode characterization of low-order TE_0_-TE_*n*_ (*n* ≤ 10) mode operator, the fabricated PIC consists of a mode multiplexer with input ports I_0_−I_*n*_, a mode operator, and a mode demultiplexer with output ports O_0_–O_*n*_. The injected TE_0_ mode from the input port *I*_*j*_(*j* = 0 and *n*) is first multiplexed to the TE_*j*_ mode (*j* = 0 and *n*) in the multimode bus waveguide, and then transmits through the metamaterial mode operator. After mode conversion, all modes are demultiplexed to the TE_0_ mode and exit the corresponding output port *O*_*k*_(*k* = 0, 1,…, *n*). The mode transmission from the TE_*j*_ mode (*j* = 0 and *n*) to the TE_*k*_ mode (*k* = 0, 1,…, *n*) is finally obtained after normalization with respect to the transmission of the reference circuit.

### High-speed data communication of 28-GBaud 16-QAM signal

Multimode silicon photonics has offered new opportunities for extensive research fields ranging from quantum photonics, topological photonics as well as nonlinear photonics. Here, we take the high-speed data transmission scenario as a proof-of-principle application of the metamaterial-assisted universal multimode manipulation. The experimental setup is given in Fig. 5d. The output wavelength of the tunable laser is set to be 1540 nm to match the peak wavelength of the grating coupler. A 28-GBaud 16-QAM signal is generated by the digital-analog converter with a sample rate of 64 GSa/s, and boosted by electronic amplifiers to drive the 22-GHz IQM. The modulated optical signal is then amplified and time gated by an acousto-optic modulator with a duty cycle of 12.5%. To emulate eight WDM channels with a spacing of 50 GHz, a spectrally shaped amplified spontaneous emission noise is generated and then combined with the time-gated signal. The obtained WDM signal is amplified and split into eight individual components, which suffer different time delays for signal decorrelation before being injected into the MDM circuit. At the receiver side, the local oscillator is gated and delayed in a similar manner. Meanwhile, polarization multiplexing and time-division multiplexing technology are utilized before the output signals detected by a polarization-diverse coherent receiver. The received electric signals are sent into a real-time oscilloscope to recover the 16-QAM data, where a multiple-in-multiple-output–based digital signal processing algorithm is adopted to mitigate the inter-CT.

## Supplementary information

Supplementary Information for Metamaterial Enabled Arbitrary on-chip Spatial Mode Manipulation
