# Supplementary material for: Metamaterial-enabled arbitrary on-chip spatial mode manipulation
Source: Light Sci Appl. 2022 Jun 1;11:168. doi: 10.1038/s41377-022-00859-9 (PMC9160251; doi:10.1038/s41377-022-00859-9)
Supplement: Supplementary file 1 — Supplementary Information for Metamaterial Enabled Arbitrary on-chip Spatial Mode Manipulation [file 41377_2022_859_MOESM1_ESM.pdf]

Supplementary Information for

# Metamaterial Enabled Arbitrary on-chip Spatial Mode Manipulation

Jinlong Xiang, Zhiyuan Tao, Xingfeng Li, Yaotian Zhao, Yu He, Xuhan Guo*, and Yikai Su*

State Key Laboratory of Advanced Optical Communication Systems and Networks, Department of Electronic Engineering, Shanghai Jiao Tong University, Shanghai 200240, China

***Corresponding author: guoxuhan@sjtu.edu.cn; yikaisu@sjtu.edu.cn**

**This file includes:**

**Note 1. Metamaterial building block concept and beam shaping principle**

**Note 2. Theoretical analysis of high-order mode operators**

**Note 3. Topology arrangement of metamaterial BBs in even-order mode operators**

**Note 4. Analysis of the fabrication tolerance and thermal sensitivity**

**Note 5. Experimental results for high-order mode operators**

**Note 6. Performance comparison of state-of-the-art mode converters**

**Note 7. Design details of the ADCs and SWGs for mode (de)multiplexing**

**Note 8. Performance enhancement of mode manipulation with properly-engineered taper**

**Note 9. $TE_0$-$TE_2$ metamaterial building block in a low index contrast platform**

**Note 10. Characterization of 8-channel MDM circuit**

## Note 1. Metamaterial building block concept and beam shaping principle

The concept of building blocks (BBs) has been a powerful design tool in both electronic engineering, e.g., the field programmable gate array (FPGA) and photonic integrated circuits (PICs), e.g., the process design kits (PDKs) released by commercial foundries. For instance, the Mach-Zehnder interferometer (MZI) mesh consisting of simple waveguides, phase shifters, and power splitters/combiners can find many important applications[1], including matrix multiplication in neuromorphic photonics, tunable filters in microwave photonics, optical beamforming in Lidar, etc. However, the state-of-the-art mode manipulation techniques are all specific mode-order-oriented[2], which require difficult initial structure selection based on tuition and extensive geometric parameter optimization for each single mode case, leading to a long development time and huge trial-and-error costs. The BBs design methodology has motivated us to build a universal framework to excite and manipulate arbitrary high-order modes in a simple yet efficient way.

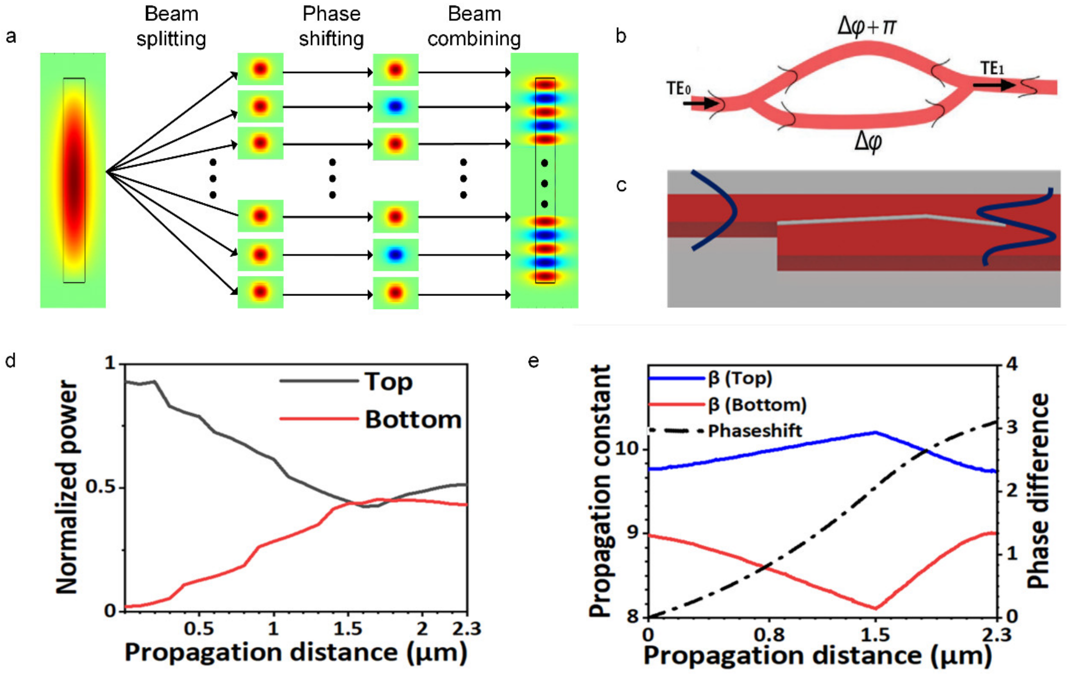


**Fig. S1 | a** Working principle of the beam shaping method. **b** Mode conversion based on the Mach-Zehnder interferometer (MZI) structure, where long optical paths are necessary to induce a phase difference of $\pi$ between two branches. **c** A single dielectric slot can function as a power splitter and a phase shifter simultaneously. **d** Normalized power in the top and bottom waveguide. **e** Accumulated phase difference between the top and bottom waveguides.

On-chip mode conversion techniques have been detailly overviewed in our previous review work[2], i.e., phase matching method, beam shaping method, constructive interference of coherent scattering, and metamaterial structures. Among them, beam shaping is the most intuitive and straightforward technique for arbitrary high-order mode conversion. Figure S1a illustrates the working principle, where the $N_{th}$ order TE mode is considered as a combination of $n+1$ antiphase adjacent quasi-$TE_0$ modes[3]. The input fundamental mode is first split into $n+1$ beams with equal power, which then travel through different effective lengths in the conversion region and achieve the antiphase condition between neighboring components of high-order mode, thus eventually combined to excite the desired $TE_n$ output mode. However, conventional demonstrations of beam shaping are typically realized with the MZI architecture (shown in Fig. S1b)[4-6], where it's inevitable to implement complicated structures for power splitting/combining and long optical paths for phase shifting. Consequently, the designs may suffer not only large footprints but

also poor scalability to high-order mode conversion (see Table S1).

Our previous study has proved that a single dielectric slot, splitting the multimode waveguide into two single-mode waveguides as shown in Fig. S1c, can efficiently function as a power splitter and a phase shifter simultaneously[7]. It can be seen that the optical power in the top and bottom waveguide are almost equal (Fig. S1d) and the accumulated phase difference between two beams is approximately π (Fig. S1e) at the end of the dielectric slot. This interesting finding has inspired us to further explore the possibility of implementing the beam shaping technique for arbitrary mode manipulator completely with metamaterial BBs. Given the symmetry property of electric mode profiles, the arrow-like nanostructure is a natural and promising primitive for the $TE_0$-$TE_2$ mode manipulation. As shown in Fig. S2a-b, the two straight arms are able to separate the multimode waveguide into three single-mode channels, and the followed V-shaped groove can serve as a phase shifter and a power combiner. We then perform numerical simulations to verify the idea and coarsely optimize the geometric parameters with the standard coupled-mode theory (CMT). The finalized BB structure is ultimately confirmed by the delicate optimization with 3D finite-difference-time-domain (FDTD solutions, Lumerical).

As for the layout arrangement of dielectric BBs for high-order mode operators, i.e., the distribution of dielectric perturbation, beam shaping method indicates that in order to separate the input mode into multiple equal beams, the $TE_0$-$TE_2$ BBs must be placed at the positions of "peaks" or "valleys" in the transverse mode profile of high-order modes. Taking the $TE_0$-$TE_6$ mode operator as an example (Fig. S2c-d), the six straight dielectric slots divide the multimode waveguide into seven single-mode channels where seven quasi-$TE_0$ modes are generated first, and the three V-shaped grooves introduce proper phase differences between adjacent beams and combine them into the $TE_0$/$TE_6$ mode. It should be noted that this kind of BBs' layout topology actually guarantees the maximum of the field overlap integral between two desired modes in the CMT model as well. To further simplify the designs of high-order mode operators, we have provided a set of mathematical formulas to quickly and accurately determine the waveguide width and BBs' positions in the main manuscript.

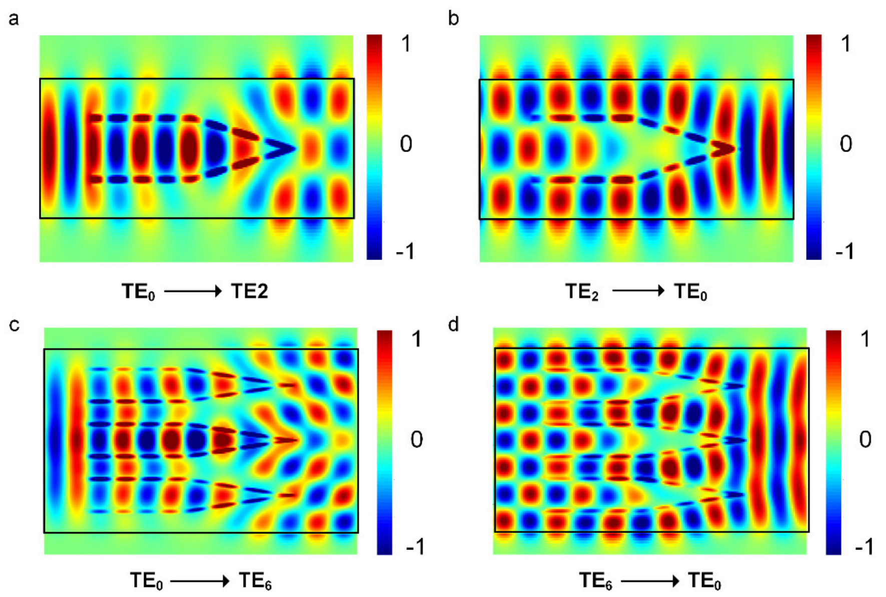


**Fig. S2** | Simulated electric field distribution of **a-b** the $TE_0$-$TE_2$ mode operator and **c-d** the $TE_0$-$TE_6$ mode operator.

## Note 2. Theoretical analysis of high-order mode operators

The mode evolution process in a perturbed waveguide can be described with the coupled mode theory (CMT). Just as explained in the main manuscript, metamaterial building blocks (BBs) are exactly introduced to positions where "peaks" and "valleys" appear in the electric field profile of desired high-order mode, as shown in the left column of Fig. S3. The calculated mode conversion efficiency for high-order mode operators as a function of the propagation distance are given in the middle column of Fig. S3, which have good quantitative agreement with the simulated results obtained from 3D-FDTD simulations. The corresponding coupling coefficients are also given in the right column of Fig. S3. It should be noted that only limited eigenmodes are considered in the theoretical model, and all potential scattering losses are ignored for simplicity. Therefore, the increase of evolved mode number in high-order mode operators will lead to larger differences between the results of CMT model and 3D-FDTD simulations.

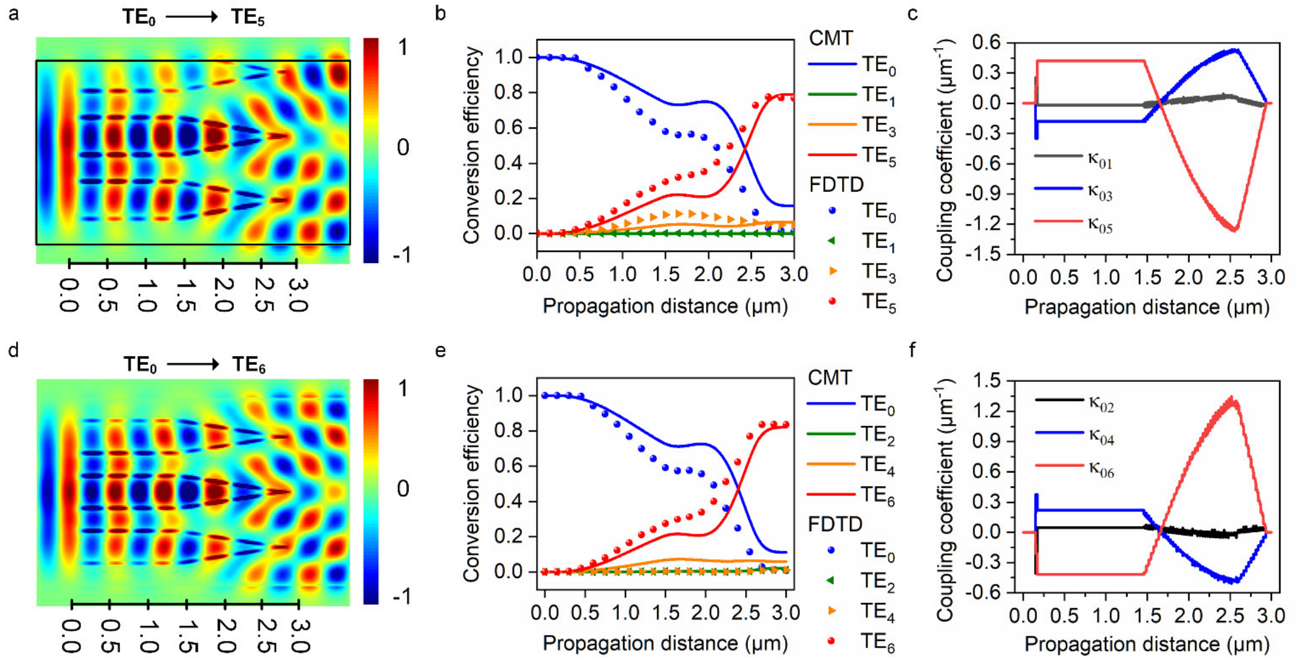


**Figure S3. Theoretical analysis of the building-block-based mode operators.** Displayed from left to right are the simulated electric field distributions at 1550 nm (left), the calculated mode conversion efficiency as a function of the propagation distance from both coupled mode theory model and 3D-FDTD (medium), and the corresponding coupling coefficients (right).

## Note 3. Topology arrangement of metamaterial BBs in even-order mode operators

As shown in Fig. S4a, there exists $N/2+1$ in-phase field "peaks" and $N/2$ anti-phase "valleys" in the transverse electric field profile of the $TE_N$ mode ($N$ is an even number), thus leading to two possible topology layouts where metamaterial BBs are pointing at either the field "valleys" or the "peaks". The former has been detailly discussed in the main manuscript, and we illustrate the latter scheme in the following content. Similar to the situations of odd-order mode operators, the two metamaterial BBs placed on both sides partially extend beyond the original waveguide region and are truncated directly. The whole structure is symmetrical and the waveguide width is determined by the same formula:

$$w_{even} = w_d \frac{N}{2} + 2w_{extra}\ (\mu m) \tag{1}$$

where $w_d = 0.92$ µm and $w_{extra} = 0.19$ µm. The simulated excess losses (ELs) and modal crosstalk (CT) as a function of the even mode order are given in Fig. S4b and Fig. S4c, respectively. With the increase of mode order, even-order mode operators finally converge to uniform performance with the ELs below 1.5 dB and CT lower than −8 dB in the wavelength range from 1500 nm to 1600 nm. It should be noted that this kind of performance consistency between two topology layouts confirms the effectiveness of the proposed metamaterial BBs-based design framework.

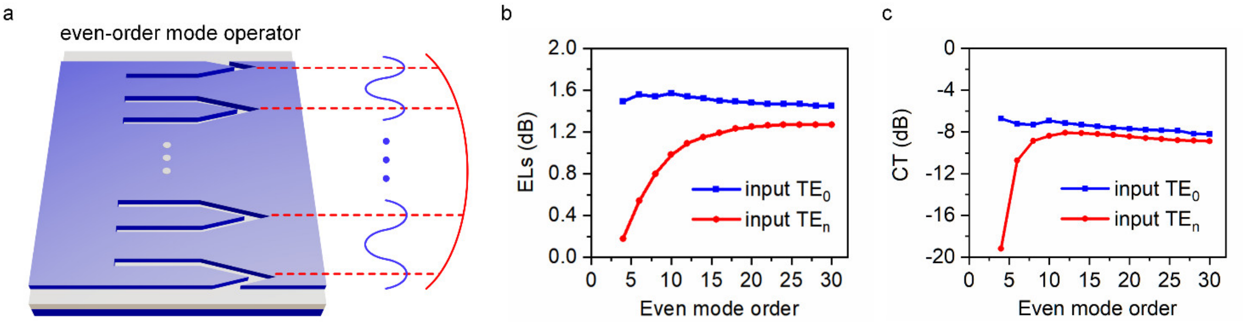


**Figure S4. Topology layout and simulated results for even-order mode operators. a** Arrangement of metamaterial BBs pointing at the $n+1$ in-phase "peaks". **b** Corresponding calculated excess losses (ELs), and **c** modal crosstalk (CT) in the wavelength range from 1500 nm to 1600 nm.

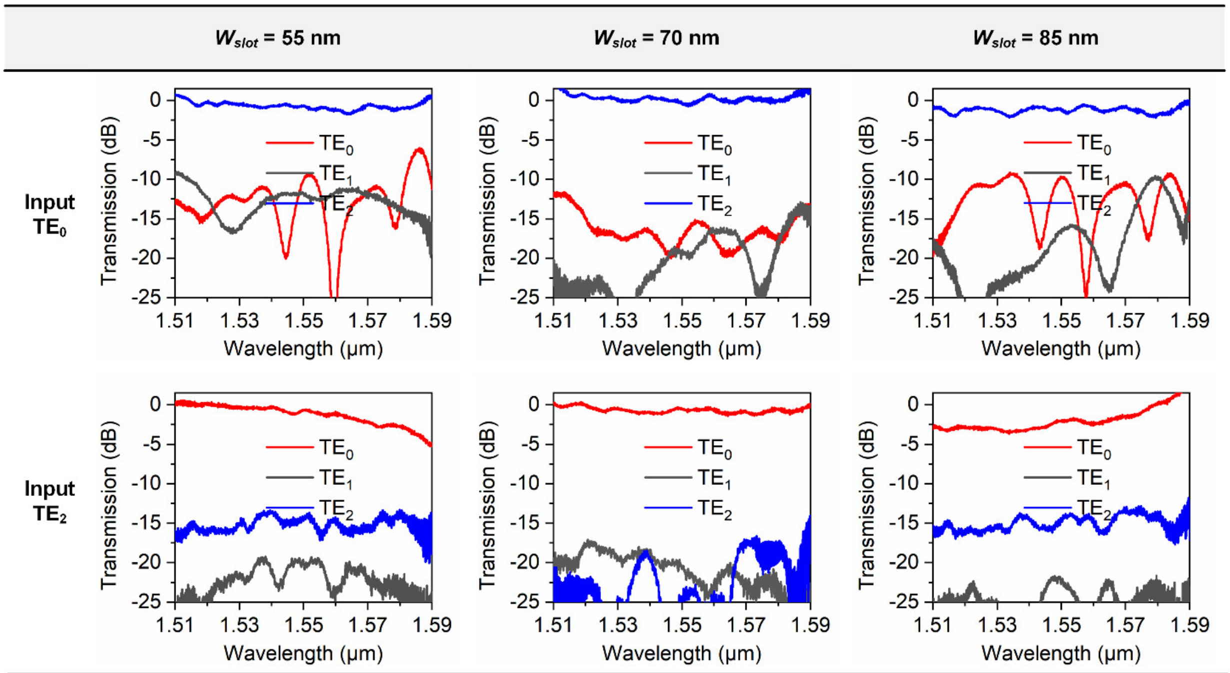


**Fig. S5** | Measured transmission spectra of the $TE_0$-$TE_2$ building block with the dielectric slot width $W_{slot}$ under variations of $\pm$ 15 nm.

## Note 4. Analysis of the fabrication tolerance and thermal sensitivity

Considering the metamaterial BBs may suffer imperfections during the fabrication process, we have experimentally investigated the device tolerance by fabricating metamaterial BBs with three different slot widths of 55 nm, 70 nm, and 85 nm. The measured transmission spectra are presented in Fig. S5. It can be seen that the BBs suffer a shift of the central operation wavelength and

exhibit performance degradation to some degree, which can be attributed to potential fabrication errors. However, acceptable performance with the ELs lower than 2.5 dB and CT below $-8$ dB can always be achieved within 40 nm wavelength range (1510-1550 nm for $W_{slot}$ = 55 nm and 1550-1590 nm for $W_{slot}$ = 85 nm). Moreover, we have numerically analyzed the device tolerance of high-order mode operators by varying the dielectric slot width of $\pm$15 nm in 3D-FDTD simulations. Without loss of generality, the simulated mode manipulation efficiency of four different mode operators is given in Fig. S6. It's clear that the ELs are lower than 2 dB and the CT is below $-8$ dB in the wavelength range from 1500 nm to 1600 nm for all the cases. Therefore, the proposed metamaterial BBs feature both eased fabrication process of single-step etching and good tolerance to fabrication variations.

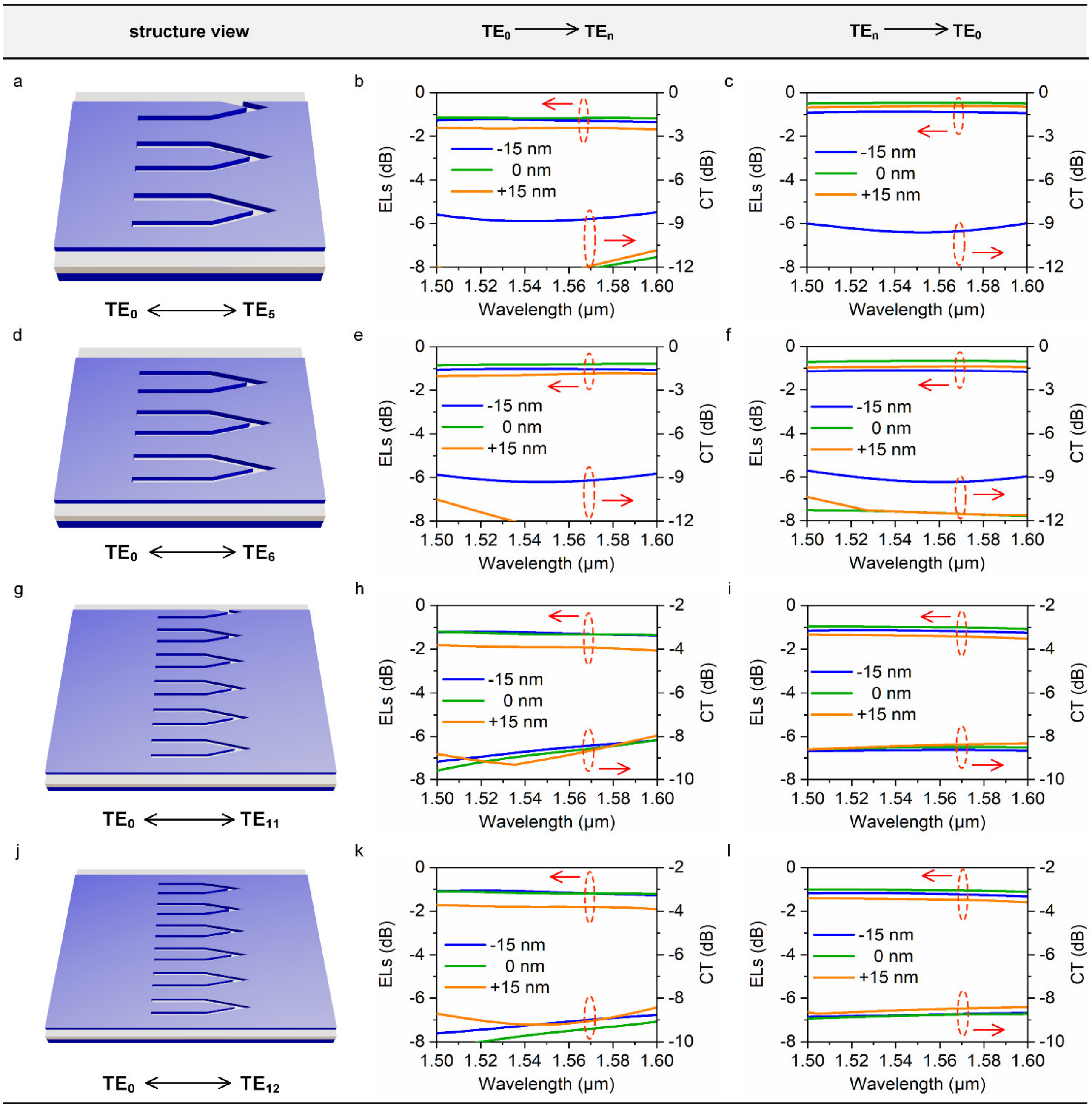


**Figure S6. Simulated transmission spectra of high-order mode operators with the dielectric slot width under variations of $\pm$15 nm.** Displayed from left to right are the schematic view of high-order mode operators (left), the simulated ELs and CT when injecting the $TE_0$ mode (medium) and the $TE_n$ mode (right).

The fluctuations of environment temperature may also influence the device performance in real integrated applications, though most of the works are demonstrated without the temperature controller for low-cost application purpose. In previous studies, the thermal sensitivity has been analyzed by simulating the mode conversion efficiency with the temperature variation of 100 K[8-11]. Here we also evaluate this impact by considering the refractive index change of silicon with $dn_{Si}/dT = 1.86 \times 10^{-4}\ \mathrm{K}^{-1}$. When the temperature changes from 300 K to 500 K, the ELs (CT) for the $TE_0$-to-$TE_2$ mode conversion only increase from 1.1 dB (−16.8 dB) to 1.23 dB (−16.25 dB) in the wavelength range of 1500 − 1600 nm (Fig. S7a). While for the $TE_2$-to-$TE_0$ mode conversion (Fig. S7b), the ELs (CT) increase from 0.36 dB (−14.3 dB) to 0.39 dB (−12.7 dB). Besides, the $TE_0$-$TE_5$ (Fig. S7c-d) and $TE_0$-$TE_{12}$ (Fig. S7e-f) mode operators exhibit nearly stable performance. It's concluded that the metamaterial mode operators are robust to temperature variations, and extra temperature control equipment is unnecessary.

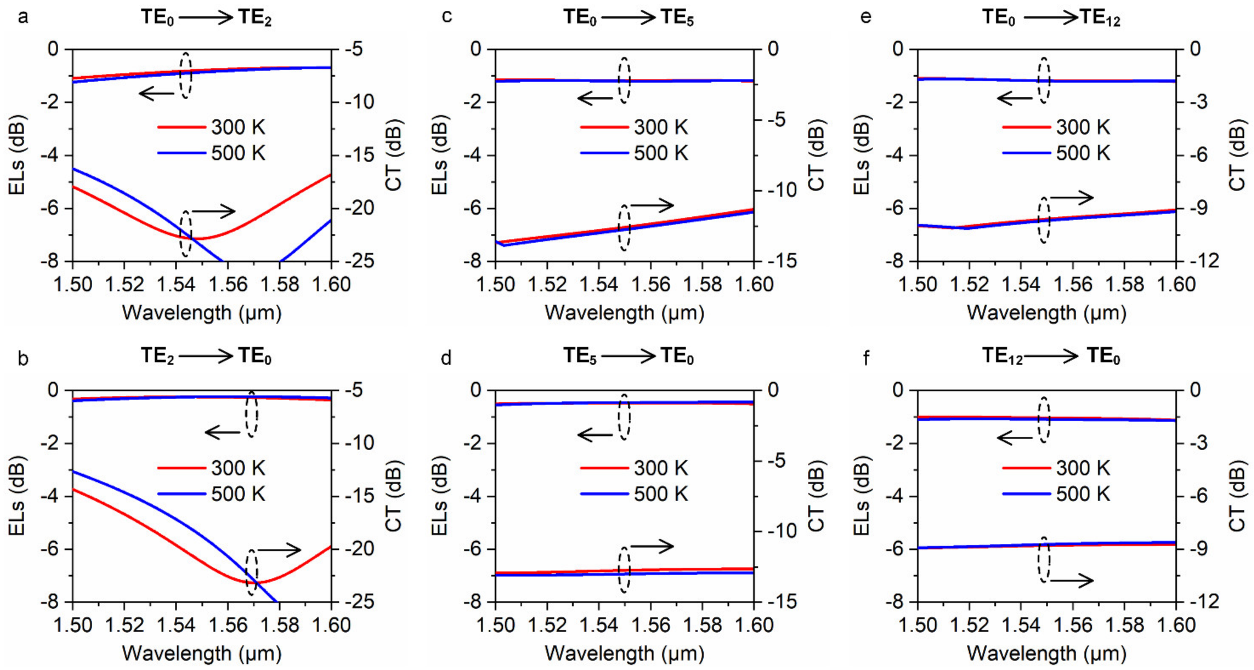


**Fig. S7** | Simulated ELs and CT under the temperature of 300 K and 500 K. **a-b** $TE_0$-$TE_2$ mode operator, **c-d** $TE_0$-$TE_5$ mode operator, **e-f** $TE_0$-$TE_{12}$ mode operator.

## Note 5. Experimental results for high-order mode operators

To experimentally verify the proposed metamaterial BBs concept, we have fabricated a set of mode operators and characterized them with the mode-by-mode method. Except for the results given in the main manuscript ($TE_0$-$TE_2$, $TE_0$-$TE_5$, and $TE_0$-$TE_{10}$), the measured mode manipulation efficiency for more high-order mode operators is provided in this section, depicted in Fig. S8. For the $TE_0$-$TE_3$ mode operator, the measured ELs are lower than 2.8 dB and the CT is below −10.1 dB in the wavelength range from 1510 nm to 1570 nm (Fig. S8**a**). Besides, the $TE_0$-$TE_4$ mode operator exhibits similar good performance with the ELs lower than 3.5 dB and the CT below −10.5 dB from 1510 nm to 1570 nm (Fig. S8**b**). As for the $TE_0$-$TE_6$ mode operator, the measured ELs are below 3.1 dB with the CT lower than −8.9 dB in the 1510 − 1580 nm wavelength band (Fig. S8**c**). Although the $TE_0$-$TE_9$ mode operator suffers performance degradation to some degree in terms of the CT (< −5.6 dB), which may be attributed to potential fabrication imperfections, its merit of ELs still maintain well (< 2.4 dB) across the wavelength band from 1530 nm to 1570 nm (Fig. S8**d**). In general, these experimental results show good agreement with the 3D-FDTD simulations.

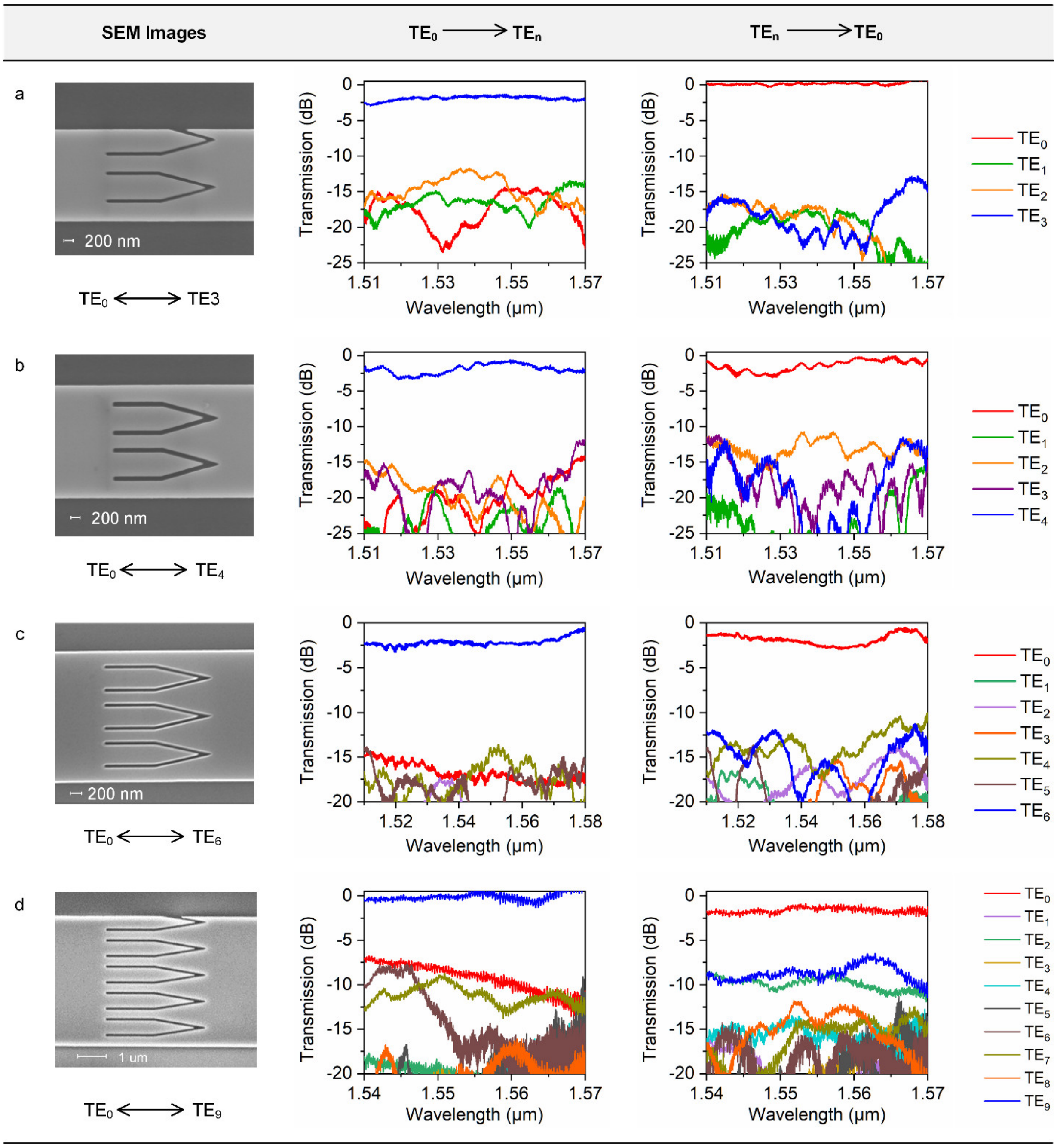


**Figure S8. Experimental results of high-order mode operators.** Displayed form left to right are the SEM image of a fabricated mode operator (left), the measured mode conversion efficiency when the input is the $TE_0$ mode (middle) and the $TE_n$ mode (right).

For the characterization of $TE_0$-$TE_n$ ($n \geq 15$) mode conversion, we have fabricated cascaded structures containing 2, 6, and 10 mode operators on the same chip. The measured transmission spectra for the cases of 2 and 10 are given in Fig S9a-b ($TE_0$-$TE_{19}$) and Fig S9d-e ($TE_0$-$TE_{20}$). Similar to the results presented in the main manuscript, here the general trends of experimental results match with the simulation results, especially for the positions and shapes of resonance dips. It should be noted that the layout arrangement of multiple back-to-back mode operators is relatively more sensitive to fabrication imperfections. As shown in Fig. S9c ($TE_0$-$TE_{19}$)) and 9f ($TE_0$-$TE_{20}$), the simulated transmission spectra of 6 cascaded mode operators can differ a lot even if the dielectric slot width is under variations of only $\pm 5$ nm, which largely explains the differences between simulation and

experimental results. Overall, the measured results match well with the simulation results for $TE_0$-$TE_{19}$/$TE_0$-$TE_{20}$ mode conversion, thus confirming the good scalability of the metamaterial BBs-enabled design framework.

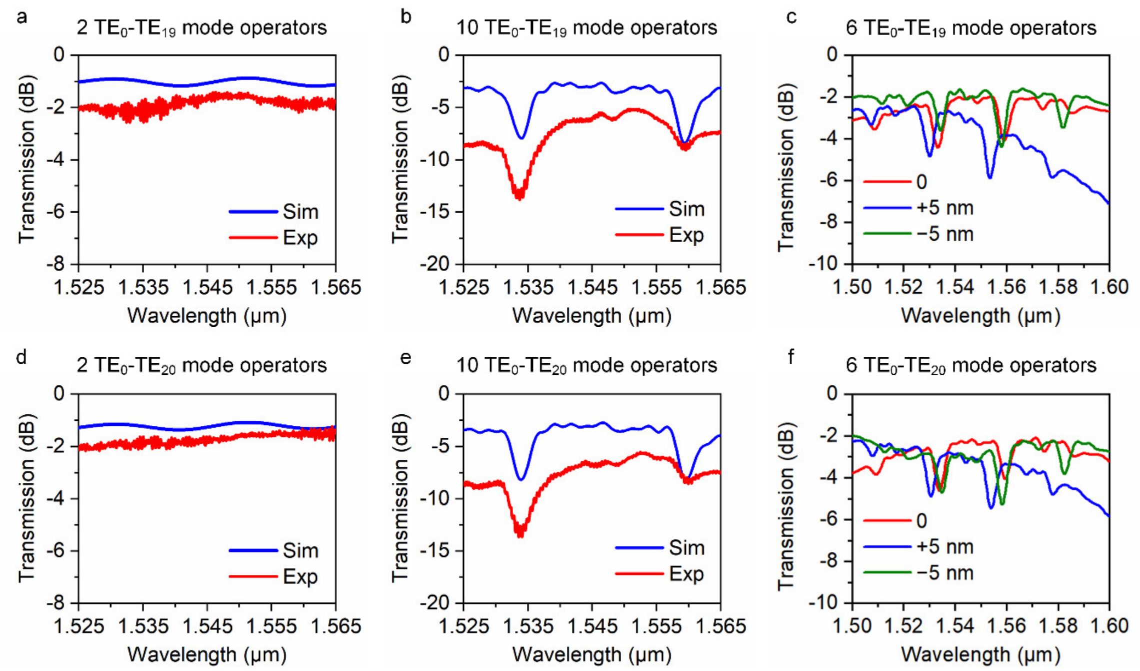


**Fig. S9** | Measured transmission spectra of cascaded structures containing **a** 2, **b** 10 $TE_0$-$TE_{19}$ mode operators. **d-e** Same results for $TE_0$-$TE_{20}$ mode operators. **c** Simulated transmission spectra with the dielectric slot width under variations of ±5 nm for 6 cascaded $TE_0$-$TE_{19}$ mode operators and **f** $TE_0$-$TE_{20}$ mode operators.

## Note 6. Performance comparison of state-of-the-art mode converters

Table S1 provides a detailed summary of state-of-the-art on-chip mode converters. It can be seen that the proposed metamaterial BBs-based design framework can not only take the full advantages of metamaterial structures, i.e., ultra-compact footprints (2.7 μm-long), broad bandwidth, but also break the long-held limitations on scalability by manipulating arbitrary high-order modes with uniform good performance. Furthermore, the BBs-based mode operators are able to implement the functionality of mode exchange, which is only supported by a small portion of mode converters reported so far.

| Working principle | Reference | Type | ELs (dB) | CT (dB) | Bandwidth (nm) | Footprint ($\mu m^2$) | Mode exchange |
|---|---|---|---|---|---|---|---|
| Phase matching | [12] | $TE_0$-$TE_{12}$ | < 1.5 | —— | 75 | 5.19 × 75 | No |
| | [13] | $TE_0$-$TE_1$ | < 0.3 | < −16 | 100 | 1.34 × 50 | No |
| | [14] | $TE_0$-$TE_{10}$ | < 2.3 | < −10 | 50 | ~14.4 × 5 | No |
| | [15] | $TE_0$-$TE_1$ | < 1.2 | < −10 | 120 | 1.83 × 62.5 | No |
| Coherent scattering | [9] | $TE_0$-$TE_1$ | ~2.2 | < −6.3 | 50 | 1.6 × 4 | Yes |
| | [10] | $TE_0$-$TE_1$ | < 2.3 | < −13.7 | 40 | 4 × 1.6 | No |
| | [16] | $TE_0$-$TE_1$ | < 1.2 | < −22 | 60 | 4.8 × 1.2 | No |

| | | | | | | | |
|---|---|---|---|---|---|---|---|
| | | $TE_0$-$TE_2$ | $< 2.5$ | $< -19$ | | | |
| | [8] | $TE_0$-$TE_2$<br>$TE_1$-$TE_3$ | $< 3.6$<br>$< 5.1$ | $< -10.7$<br>$< -13.1$ | 40 | $4 \times 3$ | No |
| | [17] | $TE_0$-$TE_1$ | $< 1$ | $< -12$ | 43 | $3.6 \times 6.3$ | No |
| Beam forming | [4] | $TE_0$-$TE_3$<br>$TE_1$-$TE_2$ | $< 1.5$ | $< -10.2$ | —— | $\sim 44 \times 25600$ | Yes |
| | [5] | $TE_0$-$TE_1$ | $< 0.4$ | —— | —— | $3 \times 18$ | No |
| | [18] | $TE_0$-$TE_1$ | $< 1$ | $< -20$ | 75 | $1.98 \times 20$ | No |
| Metamaterial | [19] | $TE_0$-$TE_1$<br>$TE_0$-$TE_2$ | $< 1$<br>$< 0.5$ | $< -10$<br>$< -10$ | 20 | $1.1 \times 5.75$<br>$1.4 \times 6.736$ | No |
| | [20] | $TE_0$-$TE_1$ | $< 0.78$ | $< -12.79$ | —— | $1 \times 23$ | Yes |
| | [21] | $TE_0$-$TE_2$ | $< 0.5$ | $< -8$ | 40 | $2 \times 7$ | No |
| | [22] | $TE_0$-$TE_3$ | $< 3.5$ | $< -11.5$ | 25 | $3 \times 16.2$ | No |
| | [23] | $TE_0$-$TE_1$<br>$TE_0$-$TE_2$ | $< 0.22$<br>$< 0.32$ | $< -12$<br>$< -13.2$ | 87 | $1.3 \times 2.7$<br>$1.9 \times 2.9$ | Yes |
| | [7] | $TE_0$-$TE_1$<br>$TE_0$-$TE_2$ | $< 0.5$<br>$< 0.3$ | $< -7$<br>$< -9$ | 50 | $0.88 \times 2.3$<br>$1.4 \times 2.4$ | No |
| | Our work | $TE_0$-$TE_2$<br>$TE_0$-$TE_3$<br>$TE_0$-$TE_4$<br>$TE_0$-$TE_5$<br>$TE_0$-$TE_6$<br>$TE_0$-$TE_9$<br>$TE_0$-$TE_{10}$<br>$TE_0$-$TE_{20}$ | $< 1.5$<br>$< 2.8$<br>$< 3.5$<br>$< 1.7$<br>$< 3.1$<br>$< 2.4$<br>$< 3.8$<br>—— | $< -15$<br>$< -10.1$<br>$< -10.5$<br>$< -8.3$<br>$< -8.9$<br>$< -5.6$<br>$< -7$<br>—— | 60<br>60<br>60<br>70<br>70<br>30<br>30<br>—— | $1.23 \times 2.7$<br>$1.76 \times 2.7$<br>$2.22 \times 2.7$<br>$2.68 \times 2.7$<br>$3.14 \times 2.7$<br>$4.52 \times 2.7$<br>$4.98 \times 2.7$<br>$9.58 \times 2.7$ | Yes |

**Table S1. Experimental performance comparison of on-chip mode converters.**

## Note 7. Design details of the ADCs and SWGs for mode (de)multiplexing

To characterize the metamaterial BBs-based mode operators with the traditional mode-by-mode method, cascaded ADCs and SWGs are employed in the experiment as the references mode (de)multiplexers, which have been verified and published from difference research groups[14,24-26]. Besides, the two-stage mode conversion strategy, enabled by metamaterial mode operators and $TE_n$-to-$TE_{n+1}$ ADCs, is utilized to multiplex high-order modes ($TE_2$, $TE_3$, …., $TE_7$) into the bus waveguide of the 8-channel MDM circuit. The design parameters of $TE_n$-to-$TE_{n+1}$ ADCs are listed in Table S2. The coupling gap is fixed to be 180 nm, and the waveguide widths are optimized to satisfy the phase matching condition between the $TE_n$ mode and the $TE_{n+1}$ mode only. Under the same effective index change, the variation range of waveguide width becomes larger as the mode order increases, as explained

in Fig. S10. Compared with the $TE_0$-to-$TE_n$ ADCs, the dispersion slopes of the bus waveguide and the access waveguide of $TE_n$-to-$TEn_{+1}$ are much closer, thus greatly releasing the device tolerance to fabrication imperfections.

| | Gap (μm) | Coupling length (μm) | Bus waveguide (μm) | Access Waveguide (μm) |
|---|---|---|---|---|
| $TE_0$ - $TE_1$ | 0.18 | 30 | 0.93 | 0.45 |
| $TE_1$ - $TE_2$ | 0.18 | 50 | 1.41 | 0.93 |
| $TE_2$ - $TE_3$ | 0.18 | 34 | 1.65 | 1.23 |
| $TE_3$ - $TE_4$ | 0.18 | 62 | 2.21 | 1.76 |
| $TE_4$ - $TE_5$ | 0.18 | 76 | 2.67 | 2.22 |
| $TE_5$ - $TE_6$ | 0.18 | 90 | 3.13 | 2.68 |
| $TE_6$ - $TE_7$ | 0.18 | 102 | 3.59 | 3.14 |

**Table S2. Design parameters of the $TE_n$-to-$TE_{n+1}$ ADCs.**

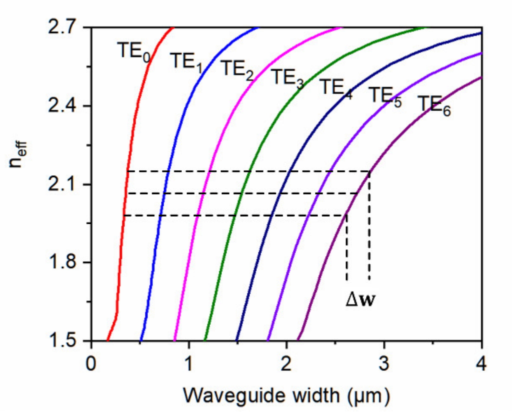


**Figure S10. Calculated effective indices of seven eigenmodes of the strip waveguide of different widths.**

## Note 8. Performance enhancement of mode manipulation with properly-engineered taper

It should be noted that the simple mathematically-defined layout arrangement of metamaterial BBs is intended to offer an initial design primitive for arbitrary high-order mode order, and the performance of mode operators is already good enough for high-speed on-chip MDM data transmission as presented in the main manuscript. Actually, the mode manipulation efficiency can be significantly enhanced by further optimizing the geometrical parameters for a specific mode order, e.g., the waveguide width, the central distance between BBs, and so on. Here we provide one of the possible optimization methods. Due to the stronger light confinement in the region of higher refractive index, the optical power of the $TE_0$ mode is mainly confined to the central waveguide region. As a result, the coupling strengths of metamaterial BBs placed on both sides actually are not as strong as those located in the center, which sets a limit to the crosstalk performance of the BBs-based design framework. To address this problem, we propose to add a properly-engineered taper ($\theta = 11.5^{\circ}$ and $L = 7$ μm) in front of the metamaterial mode operators (see Fig. S11a). As shown in Fig. S11b-c, the taper will gradually squeeze the $TE_0$ mode from the input waveguide into the narrower metamaterial waveguide. Consequently, more mode energy will be distributed to both sides of the waveguide, thus balancing the overall distribution of BBs' coupling strengths. In this way, the simulated crosstalk can be improved from $-8$ dB to $-12.5$ dB for all high-

order mode operators (see Fig. S11d-i). We note that the device occupies a larger footprint and don't support the mode exchange functionality any more in this optimization approach.

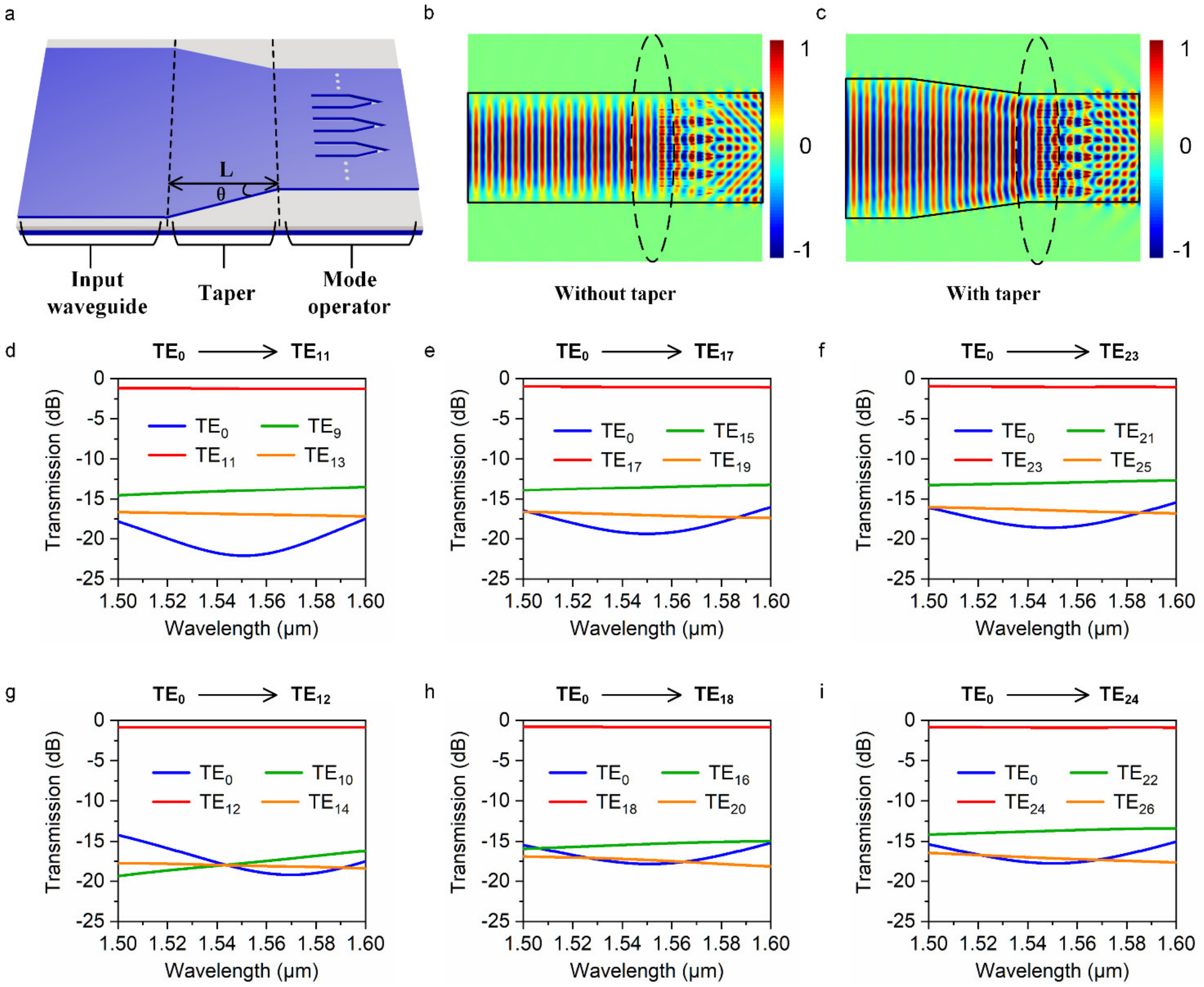


**Figure S11. Performance enhancement of properly-engineered taper. a** Schematic view of the taper-added design. **b-c** Simulated electric field distribution of the $TE_0$-to-$TE_{12}$ mode conversion without and with taper added. **d-i** Simulated $TE_0$-to-$TE_n$ mode conversion efficiency by adding a taper ($\theta = 11.5^{\circ}$ and $L = 7$ μm) in front of the BBs-based mode operators.

## Note 9. $TE_0$-$TE_2$ metamaterial building block in a low index contrast platform

The metamaterial building block is originally inspired from the beam shaping method and theoretically analyzed with the CMT model. The working principle is fully compatible with different material platforms and can be flexibly transferred to other wavelength bands. As a proof-of-concept demonstration, we have designed a $TE_0$-$TE_2$ mode operator operating in the O-band with similar arrow-like perturbation in a low-index contrast polymer platform. In simulation, we consider the waveguide having a refractive index of $n_{wg} = 1.54$ (e.g., the electron-beam resist ZEP520) surrounded by a background index of $n_{bg} = 1.34$ (e.g., the spin-on fluoropolymer CYTOP). Figure S12 presents the final optimized geometric parameters (Fig. S12a), simulated electric field distributions of two input cases (Fig. S12b and 12d), and corresponding transmission spectra (Fig. S12c and 12e). It can be seen that the ELs for the $TE_0$-to-$TE_2$ mode conversion are lower than 0.486 dB with the CT below $-12.16$ dB in the wavelength range

of 1260 − 1360 nm. While for the $TE_2$–to-$TE_0$ mode conversion, the ELs are lower than 0.484 dB with the CT below −13.46 dB in the same wavelength band. It's worth mentioning that the polymer $TE_0$-$TE_2$ building block occupies a larger device footprint compared to that in the silicon-on-insulator (SOI) platform, which can be explained by the smaller dielectric perturbation term $\Delta\varepsilon(x, y, z)$ of the coupling coefficient $\kappa_{mn}(z) = \frac{\omega}{4}\iint \psi_m^*(x, y)\Delta\varepsilon(x, y, z)\psi_n(x, y)dxdy$.

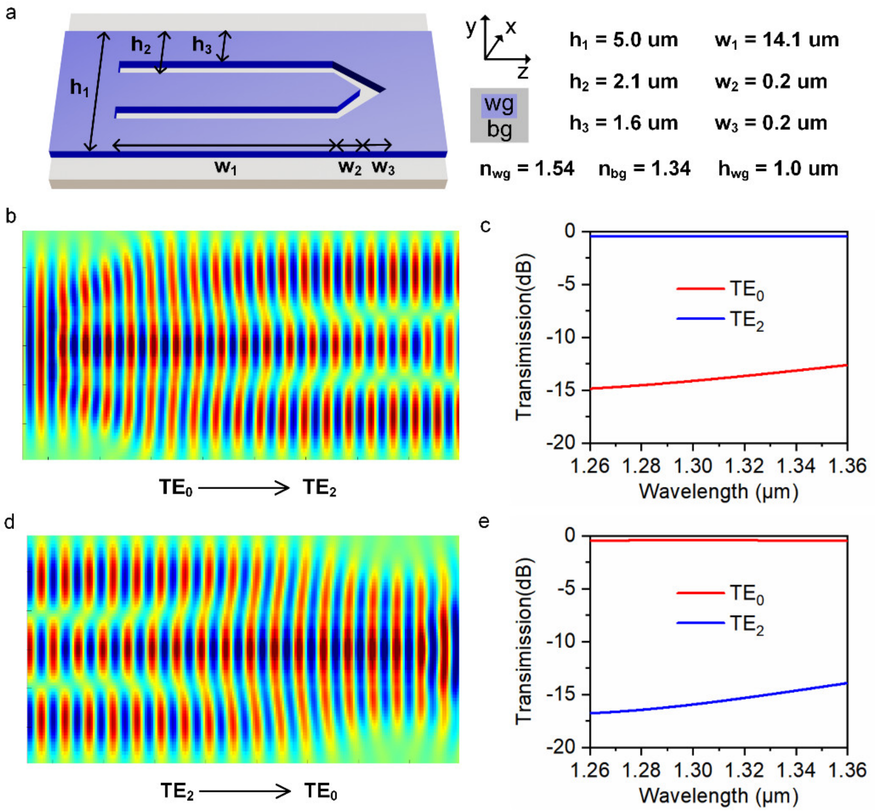


**Fig. S12** | Metamaterial $TE_0$-$TE_2$ mode operator in a low refractive index contrast system. **a** Schematic view and geometric parameters of the design. Simulated electric field distribution **b (d)** and transmission spectra **c (e)** for the $TE_0$ ($TE_2$) mode. The mode crosstalk of $TE_1$ is below −25 dB, thus not shown in both figures.

## Note 10. Characterization of 8-channel MDM circuit

A fiber array is used to couple light into and out of the silicon chip. Each time we inject light into a selected input port and record the optical signals at all output ports. Figure S13 shows the measured transmission spectra, which are normalized to the transmission of reference grating couplers fabricated on the same chip. Due to the fabrication variations, the central operation wavelength of each mode channel is different. It can be seen that the ELs vary between 0.2 dB ~ 10.1 dB and the CT ranges from −7.76 dB to −28.8 dB for all the 8 channels at 1540 nm. Although the two-stage mode multiplexing inevitably suffers much larger ELs, when the operation wavelength is properly chosen, it can still break the limitations of achievable mode order in current multiplexing schemes, thus significantly increasing the transmission capacity of on-chip MDM optical interconnects.

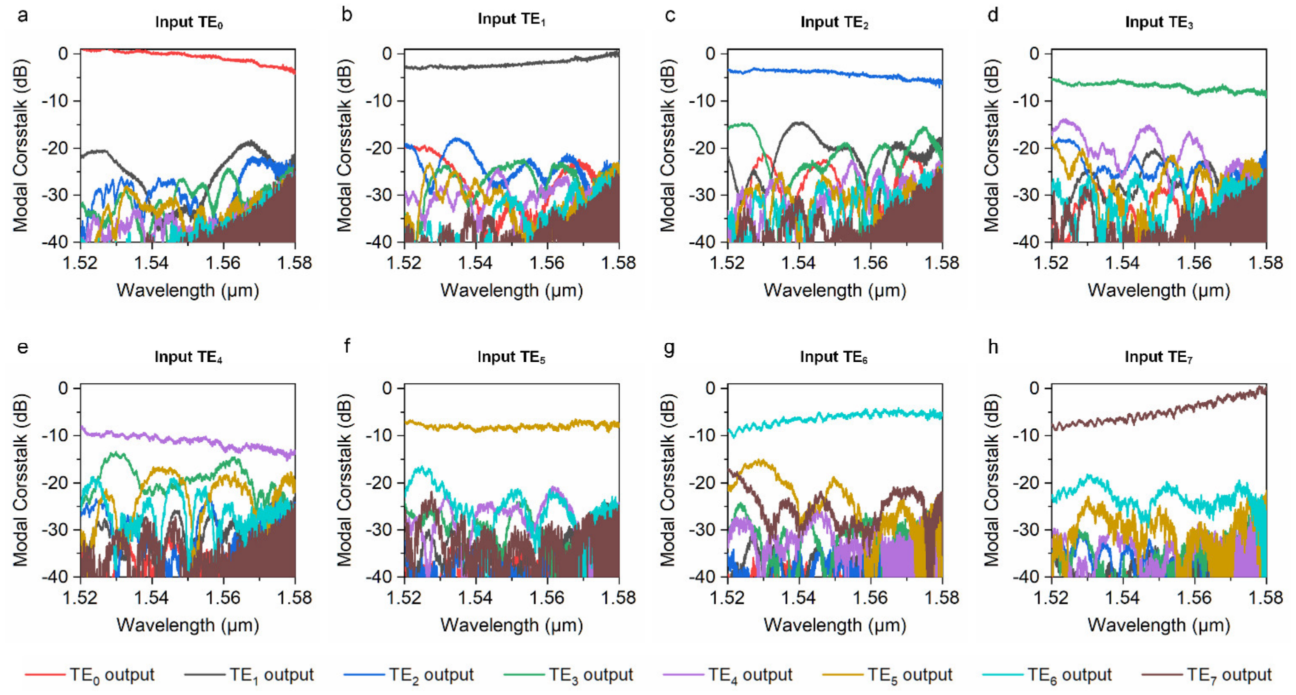


**Figure S13. Measured transmission response of the 8-channel on-chip MDM transmission.**

## References


1 Bogaerts, W. *et al.* Programmable photonic circuits. *Nature* **586**, 207-216, doi:10.1038/s41586-020-2764-0 (2020).

2 Su, Y., Zhang, Y., Qiu, C., Guo, X. & Sun, L. Silicon Photonic Platform for Passive Waveguide Devices: Materials, Fabrication, and Applications. *Adv Mater Technol-Us* **5**, 1901153, doi:https://doi.org/10.1002/admt.201901153 (2020).

3 Stern, B., Ji, X., Okawachi, Y., Gaeta, A. L. & Lipson, M. Battery-operated integrated frequency comb generator. *Nature* **562**, 401-405, doi:10.1038/s41586-018-0598-9 (2018).

4 Lee, B.-T. & Shin, S.-Y. Mode-order converter in a multimode waveguide. *Opt. Lett.* **28**, 1660-1662, doi:10.1364/ol.28.001660 (2003).

5 Huang, Y., Xu, G. & Ho, S.-T. An Ultracompact Optical Mode Order Converter. *IEEE Photonics Technol. Lett.* **18**, p.2281-2283, doi:10.1109/Lpt.2006.884886 (2006).

6 Lu, L., Liu, D., Yan, M. & Zhang, M. On-chip reconfigurable mode converter based on cross-connected subwavelength Y-junctions. *Photon. Res.* **9**, 43-48.

7 Zhao, Y. *et al.* Ultra-compact silicon mode-order converters based on dielectric slots. *Opt. Lett.* **45**, 3797-3800, doi:10.1364/OL.391748 (2020).

8 Jia, H. *et al.* Multi-channel Parallel Silicon Mode-order Converter for Multimode On-chip Optical Switching. *IEEE J. Sel. Top. Quantum Electron.* (2019).

9 Jia, H., Zhou, T., Fu, X., Ding, J. & Yang, L. Inverse-design and demonstration of ultracompact silicon meta-structure mode exchange device. *Acs Photonics* **5**, 1833-1838, doi:10.1021/acsphotonics.8b00013 (2018).

10 Jia, H. *et al.* Ultra-compact dual-polarization silicon mode-order converter. *Opt. Lett.* **44**, 4179-4182, doi:10.1364/OL.44.004179 (2019).

11 Xie, H. *et al.* Highly Compact and Efficient Four-Mode Multiplexer Based on Pixelated Waveguides. *IEEE Photonics Technol. Lett.* **32**, 166-169, doi:10.1109/LPT.2020.2964308 (2020).

12 Dave, U. D. & Lipson, M. in *Conference on Lasers and Electro-Optics.* SM3J.6 (Optical Society of America).

13 Ding, Y. *et al.* On-chip two-mode division multiplexing using tapered directional coupler-based mode multiplexer and demultiplexer. *Opt. Express* **21**, 10376-10382, doi:10.1364/OE.21.010376 (2013).

14 He, Y. *et al.* Silicon High-Order Mode (De)Multiplexer on Single Polarization. *J. Lightwave Technol.* **36**, 5746-5753, doi:10.1109/JLT.2018.2878529 (2018).

15 Sun, L., Hu, R., Zhang, Z., He, Y. & Su, Y. Ultrabroadband Power Coupling and Mode-Order Conversion Based on Trapezoidal Subwavelength Gratings. *IEEE J. Sel. Top. Quantum Electron.*, 1-1, doi:10.1109/JSTQE.2021.3092383 (2021).

16 Chang, W. *et al.* Ultra-compact mode (de) multiplexer based on subwavelength asymmetric Y-junction. *Opt. Express* **26**, 8162-, doi:10.1364/OE.26.008162 (2018).

17 Frandsen, L. H. *et al.* Topology optimized mode conversion in a photonic crystal waveguide fabricated in silicon-on-insulator material. *Opt. Express* **22**, 8525-8532, doi:10.1364/OE.22.008525 (2014).

18 Sun, C., Yu, Y., Ye, M., Chen, G. & Zhang, X. An ultra-low crosstalk and broadband two-mode (de)multiplexer based on adiabatic couplers. *Sci. Rep.* **6**, 38494, doi:10.1038/srep38494 (2016).

19 Wang, H. *et al.* Compact Silicon Waveguide Mode Converter Employing Dielectric Metasurface Structure. *Adv Opt Mater* **7**, 1801191, doi:10.1002/adom.201801191 (2018).

20 Ohana, D., Desiatov, B., Mazurski, N. & Levy, U. Dielectric Metasurface as a Platform for Spatial Mode Conversion in Nanoscale Waveguides. *Nano Lett.* **16**, 7956-7961, doi:10.1021/acs.nanolett.6b04264 (2016).

21 Hao, L. *et al.* Experimental Demonstration of Compact Mode Converter Based on Conformal Dielectric Metasurface. *IEEE Photonics Technol. Lett.* **32**, 1143-1146, doi:10.1109/LPT.2020.3013923 (2020).

22 Yao, C. *et al.* On-Chip Multi-Mode Manipulation via 2D Refractive-Index Perturbation on a Waveguide. *Adv Opt Mater* **n/a**, 2000996, doi:10.1002/adom.202000996 (2020).

23 Guo, J. *et al.* Ultra-Compact and Ultra-Broadband Guided-Mode Exchangers on Silicon. *Laser & Photonics Reviews* **n/a**, 2000058, doi:10.1002/lpor.202000058 (2020).

24 Daoxin *et al.* 10-Channel Mode (de)multiplexer with Dual Polarizations. *Laser & Photonics Reviews* (2017).

25 Wang, J., He, S. & Dai, D. On-chip silicon 8-channel hybrid (de)multiplexer enabling simultaneous mode- and polarization-division-multiplexing. *Laser & Photonics Reviews* **8**, L18-L22, doi:10.1002/lpor.201300157 (2014).

26 Miller, S. A. *et al.* Large-scale optical phased array using a low-power multi-pass silicon photonic platform. *Optica* **7**, 3-6, doi:10.1364/OPTICA.7.000003 (2020).
